# Isolated add(12)(p13) in a BCR::ABL1-Negative Neutrophilic Myeloproliferative Neoplasm Lacking Canonical Driver Mutations: A Case Report

**DOI:** 10.7759/cureus.117196

**Published:** 2026-09-30

**Authors:** Mahendra Dhaka, Pyrus Bhellum, Arjun Kachhwaha, Anjali Chimnani, Rakesh Kumar

**Affiliations:** 1 General Medicine, All India Institute of Medical Sciences, Jodhpur, IND; 2 Clinical Hematology, All India Institute of Medical Sciences, Jodhpur, IND; 3 Pathology and Lab Medicine, All India Institute of Medical Sciences, Jodhpur, IND

**Keywords:** add(12)(p13), bcr::abl1, chronic neutrophilic leukemia, csf3r, etv6, myeloproliferative neoplasm

## Abstract

Persistent neutrophilic leukocytosis with hepatosplenomegaly raises suspicion for chronic neutrophilic leukemia (CNL) and other BCR::ABL1-negative myeloid neoplasms, but diagnosis can be challenging when canonical driver mutations such as colony-stimulating factor 3 receptor (CSF3R) are absent. We report a 53-year-old man with persistent leukocytosis (total leukocyte count 64.7 × 10⁹/L) with marked neutrophilia, left-shifted granulopoiesis, and hepatosplenomegaly. BCR::ABL1 polymerase chain reaction (PCR) was negative. Bone marrow examination showed a hypercellular marrow with granulocytic predominance and a normal leukocyte alkaline phosphatase score. Comprehensive next-generation sequencing (NGS) identified no pathogenic variants in CSF3R, Janus kinase 2 (JAK2), calreticulin (CALR), myeloproliferative leukemia protein (MPL), SET binding protein 1 (SETBP1), additional sex combs-like 1 (ASXL1), KIT proto-oncogene (KIT), FMS-like tyrosine kinase 3 (FLT3), nucleophosmin 1 (NPM1), or tumor protein p53 (TP53). Conventional cytogenetics revealed a 46,XY,add(12)(p13) karyotype; fluorescence in situ hybridization (FISH) for ETS variant transcription factor 6 (ETV6) rearrangement was negative. The findings were considered consistent with a BCR::ABL1-negative neutrophilic myeloid neoplasm with an indeterminate classification in the absence of a defining molecular abnormality. The isolated ETV6-negative add(12)(p13) abnormality represents an unusual cytogenetic finding, although its pathogenic significance remains uncertain because the additional genetic material was not identified. The case underscores the value of integrating clinical, morphologic, cytogenetic, and molecular findings in diagnostically challenging neutrophilic myeloid neoplasms.

## Introduction

Persistent neutrophilic leukocytosis may reflect a reactive process, infection, chronic inflammation, malignancy, drug exposure, or an underlying clonal myeloid neoplasm [1]. Distinguishing these entities requires integration of clinical, morphological, cytogenetic, and molecular findings [1-3]. Chronic neutrophilic leukemia (CNL) is a rare BCR::ABL1-negative myeloproliferative neoplasm characterized by sustained neutrophilic leukocytosis, hepatosplenomegaly, and a hypercellular bone marrow with granulocytic proliferation, after exclusion of other myeloid neoplasms and reactive causes [2,4]. Identification of activating CSF3R mutations has substantially improved diagnostic precision and is incorporated into the current World Health Organization (WHO) and International Consensus Classification (ICC) diagnostic frameworks [5-7].

However, a subset of patients with a CNL-like clinical and morphologic phenotype may lack detectable CSF3R mutations. In such cases, the diagnosis becomes more challenging and requires careful assessment of the peripheral blood and bone marrow morphology, exclusion of reactive causes, exclusion of other myeloid neoplasms, and integration of molecular and cytogenetic findings.

The major differential diagnoses in patients presenting with persistent neutrophilic leukocytosis include CNL, atypical chronic myeloid leukemia (aCML), chronic myelomonocytic leukemia (CMML), and myeloproliferative neoplasm, unclassifiable (MPN-U). CNL is characterized predominantly by mature neutrophilia, whereas aCML is characterized by increased granulocytic immaturity accompanied by prominent dysgranulopoiesis. CMML requires persistent monocytosis, while MPN-U may be considered when features of a myeloproliferative neoplasm are present but the criteria for a specific entity are not fulfilled [2-4,8].

Structural abnormalities involving chromosome 12p13, particularly those involving the ETS variant transcription factor 6 (ETV6; TEL) locus, have been described in several hematologic malignancies, including acute leukemias, myelodysplastic syndromes, and myeloid neoplasms [9-12]. ETV6 is a transcription factor involved in normal hematopoiesis and has been implicated in leukemogenesis [10,13].

The significance of an isolated add(12)(p13) abnormality without an identifiable ETV6 rearrangement in a neutrophilic myeloid neoplasm remains uncertain. To our knowledge, an isolated ETV6-negative add(12)(p13) abnormality has not previously been reported in a CSF3R-negative neutrophilic myeloid neoplasm.

We report a diagnostically challenging BCR::ABL1-negative neutrophilic myeloid neoplasm with an isolated add(12)(p13) abnormality, negative ETV6 rearrangement testing, and no identifiable canonical driver mutation. This case illustrates the diagnostic limitations encountered when a patient has a CNL-like phenotype but lacks a defining molecular abnormality and highlights the complementary role of clinical, morphologic, molecular, and cytogenetic assessment.

## Case presentation

A 53-year-old man presented with persistent neutrophilic leukocytosis for 6 months, which was initially identified during routine medical investigations. He denied fever, recent infection, or relevant drug exposure. No significant constitutional symptoms were reported, and no relevant medical or family history was identified. On examination, the splenic tip was palpable. Initial investigations revealed a hemoglobin of 11 g/dL, total leukocyte count of 68.20 × 10⁹/L, and platelet count of 150 × 10⁹/L (Table 1).

**Table 1:** Complete laboratory profile Hb - hemoglobin; TLC - total leukocyte count; DLC - differential leukocyte count; N - neutrophils; L - lymphocytes; M - monocytes; Hct - hematocrit; MCV - mean corpuscular volume; fL - femtoliter

Parameter	Day 1	Day 30	Day 90	Normal range
Hb (g/dL)	11.0	11.7	12.7	13-17
TLC (×10⁹/L)	68.2	32.86	10.12	4-11
DLC - N/L/M (%)	84.9 / 5.9 / 5.1	90 / 5 / 4	86.0 / 4.9 / 5.8	N: 40-75 / L: 20-45 / M: 2-10
Hct (%)	33.6	-	36.6	40-50
MCV (fL)	94.9	-	95.3	80-100
Platelet count (×10⁹/L)	150	138	207	150-450

Peripheral blood smear showed marked neutrophilia with left-shifted granulopoiesis, including promyelocytes, myelocytes, metamyelocytes, and band forms, with no significant basophilia. The immature granulocyte fraction comprised 11% of circulating leukocytes, consisting of promyelocytes (4%), myelocytes (3%), and metamyelocytes (4%). No significant dysgranulopoiesis was identified.

Bone marrow examination showed a markedly hypercellular marrow (90-95% cellularity) with granulocytic predominance, no excess blasts, and no significant reticulin fibrosis (Figure 1).

**Figure 1:**
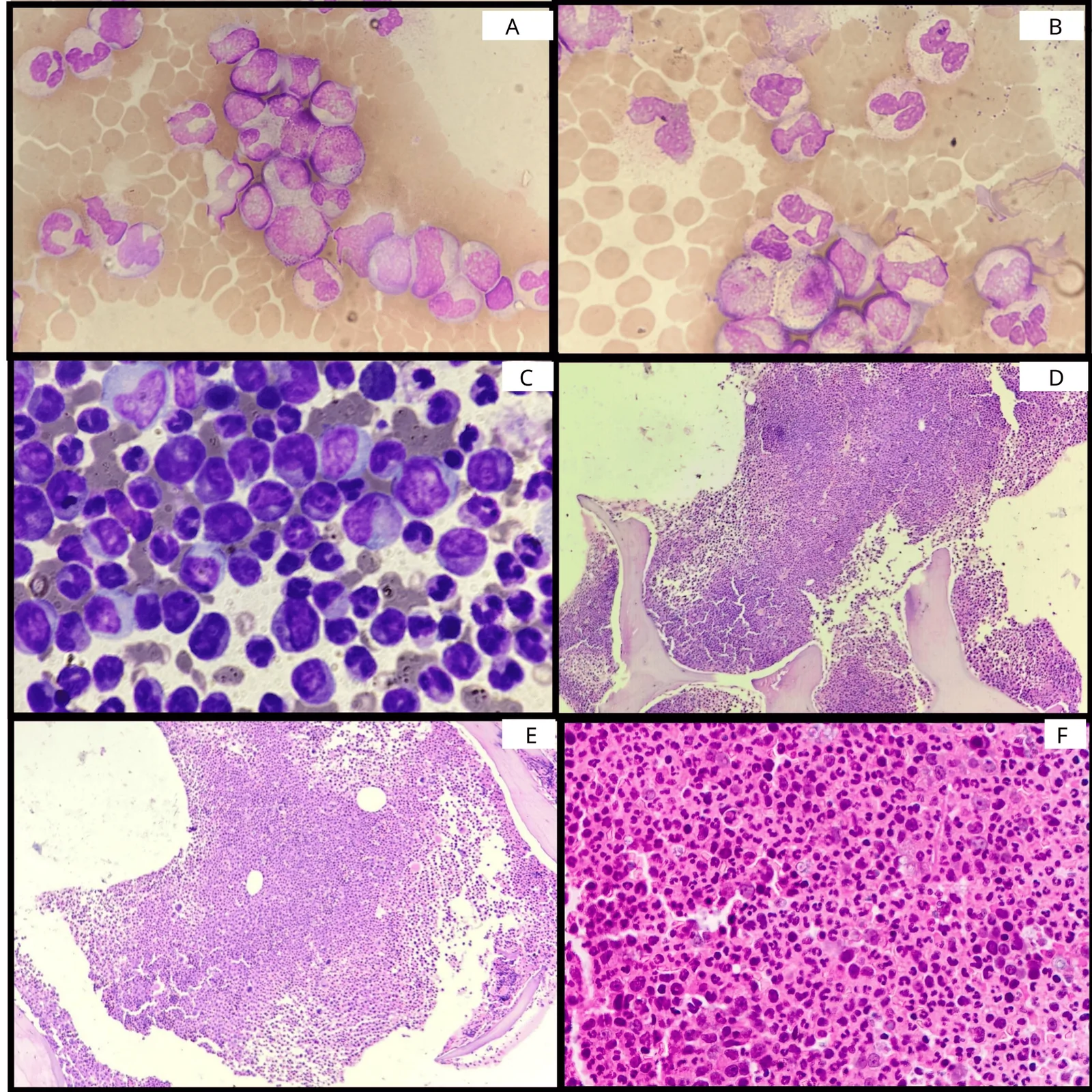
Bone marrow examination A), B) Peripheral blood smear showing leukocytosis with left shift in the granulocytic series comprising of a few neutrophils, myelocytes, and metamyelocytes (Leishman stain, 100×); C) Bone marrow aspirate revealing increased myeloid precursors with relative paucity of erythroids (Giemsa stain, 100×); D), E) Trephine biopsy showing hypercellular marrow spaces with myeloid and megakaryocytic prominence (H&E stain, 40×); F) High-power view highlighting marked prominence of granulocytes and precursors (H&E stain, 100×).

BCR::ABL1 PCR was negative, and the leukocyte alkaline phosphatase (LAP) score was 65. Contrast-enhanced CT of the abdomen confirmed hepatosplenomegaly with no other abnormality. An extensive evaluation for secondary causes of neutrophilia was performed. Evaluation for infectious, inflammatory, autoimmune, and other occult secondary causes was unrevealing, and there was no clinical or laboratory evidence of an underlying reactive process. A comprehensive next-generation sequencing (NGS) panel for myeloid neoplasms, including CSF3R, Janus kinase 2 (JAK2) (including exon 12), calreticulin (CALR), myeloproliferative leukemia protein (MPL), SET binding protein 1 (SETBP1), additional sex combs-like 1 (ASXL1), KIT proto-oncogene (KIT), FMS-like tyrosine kinase 3 (FLT3), nucleophosmin 1 (NPM1), and tumor protein p53 (TP53) identified no pathogenic variants.

Conventional cytogenetic analysis showed a 46,XY,add(12)(p13) karyotype (Figure 2). Fluorescence in situ hybridization (FISH) for ETV6 rearrangement was negative.

**Figure 2:**
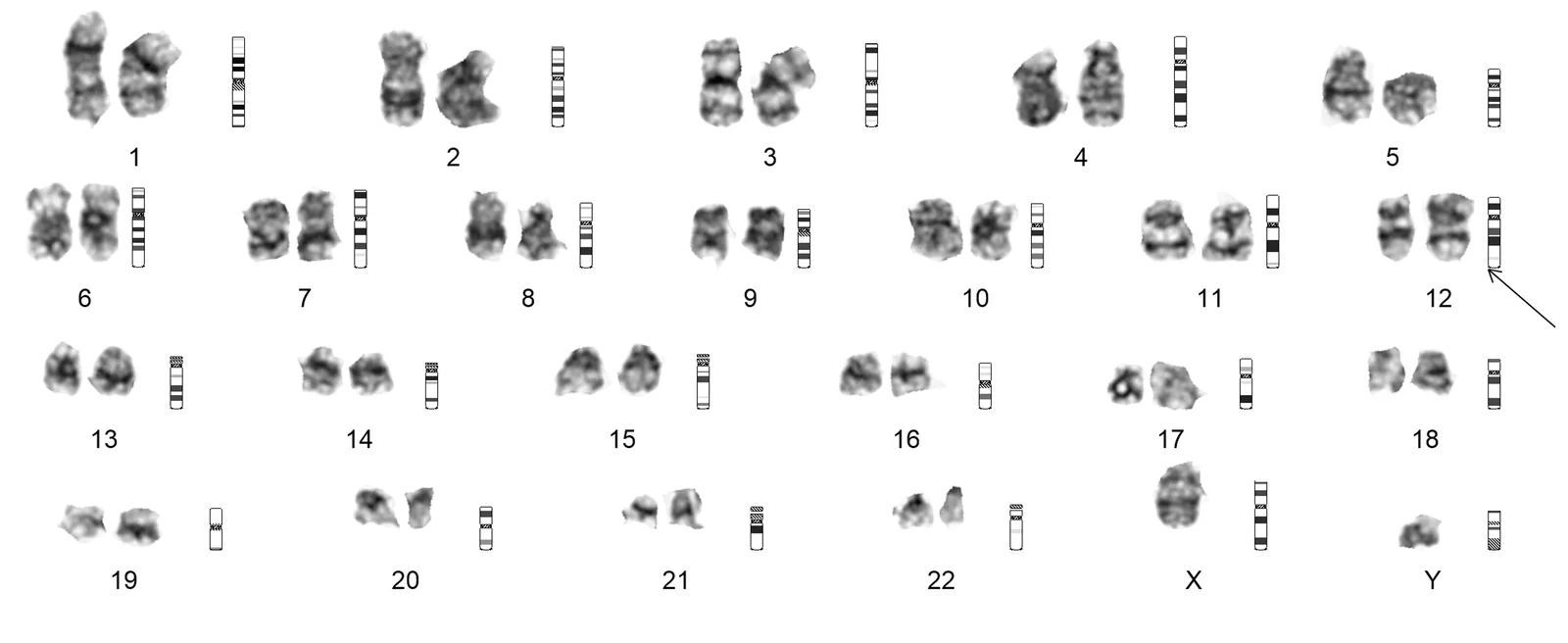
Conventional cytogenetic analysis (GTG-banding) showing a 46,XY,add(12)(p13) karyotype, with additional unidentified genetic material on the short arm of chromosome 12 at band p13 (arrow).

The overall clinical and morphologic findings suggested a chronic neutrophilic myeloid neoplasm. However, the absence of a CSF3R mutation and other canonical driver mutations prevented assignment of a definitive molecularly defined diagnosis of CNL. The differential diagnosis included CNL, atypical chronic myeloid leukemia (aCML), chronic myelomonocytic leukemia (CMML), and myeloproliferative neoplasm, unclassifiable (MPN-U). The absence of significant dysgranulopoiesis and monocytosis argued against aCML and CMML, respectively.

Although the immature granulocyte fraction was 11%, the absence of significant dysgranulopoiesis and the overall morphologic pattern were not typical of aCML. The clinical and morphologic findings were therefore considered more compatible with a neutrophilic myeloid neoplasm. The patient was started on oral hydroxyurea 500 mg twice daily, with satisfactory control of leukocytosis over the next three months. The total leukocyte count decreased from 68.2 × 10⁹/L at presentation to 32.86 × 10⁹/L at day 30 and 10.12 × 10⁹/L at day 90 (Table 1).

## Discussion

Persistent neutrophilic leukocytosis poses a diagnostic challenge, as it may reflect either a reactive process or a clonal myeloid neoplasm [1]. The differentials in our patient included CNL, MPN-U, aCML, CMML, and reactive neutrophilia secondary to an occult inflammatory or gastrointestinal process [2-4]. Several features favored a chronic myeloid neoplasm: marked leukocytosis exceeding 60 × 10⁹/L, hepatosplenomegaly, a hypercellular marrow with granulocytic predominance, and an abnormal karyotype [2,4]. An extensive evaluation for infectious, inflammatory, autoimmune, and other secondary causes of neutrophilia was unrevealing, further reducing the likelihood of a reactive process.

aCML was considered because of the left-shifted granulopoiesis. The immature granulocyte fraction was 11%, comprising promyelocytes 4%, myelocytes 3%, and metamyelocytes 4%. However, there was no significant dysgranulopoiesis, which is an important morphologic feature of aCML. Therefore, despite the degree of left shift, the overall morphologic findings were less supportive of aCML. The absence of a SETBP1 mutation provided additional, although not independently exclusionary, information. CMML was also considered but was not supported by the absence of absolute monocytosis [3]. The absence of significant basophilia and the negative BCR::ABL1 PCR further reduced the likelihood of BCR::ABL1-positive chronic myeloid leukemia.

The possibility of CNL was particularly relevant because of the marked neutrophilic leukocytosis, hepatosplenomegaly, and granulocytic-predominant hypercellular marrow. The discovery of activating CSF3R mutations has substantially refined CNL diagnostic criteria [5,13], and both the WHO 5th edition and ICC incorporate CSF3R mutation status into their diagnostic frameworks [6,7]. In the present case, however, comprehensive NGS did not identify a pathogenic CSF3R mutation. Although CSF3R-negative cases with a CNL-like phenotype have been described, the absence of this defining molecular abnormality introduces substantial diagnostic uncertainty. Therefore, the clinical and morphologic findings were considered suggestive of a neutrophilic myeloid neoplasm, but were not considered sufficient to establish a definitive diagnosis of CNL.

A comprehensive NGS panel also failed to identify pathogenic variants in JAK2, CALR, MPL, SETBP1, ASXL1, KIT, FLT3, NPM1, or TP53. These findings indicate that several recognized molecular abnormalities were not detected but do not, by themselves, exclude a clonal myeloid disorder.

A notable finding in our patient was the isolated 46,XY,add(12)(p13) karyotype. Abnormalities involving 12p13 have been reported across a range of hematologic malignancies, including acute lymphoblastic leukemia, myelodysplastic syndromes, and myeloid neoplasms [9-11]. These abnormalities most commonly involve the ETV6 locus. ETV6 is a transcription factor involved in normal hematopoiesis and has been implicated in leukemogenesis [10,14]. FISH for ETV6 rearrangement was negative in our patient. Thus, the cytogenetic finding did not demonstrate an ETV6 rearrangement by the assay performed. Importantly, conventional karyotyping identified additional genetic material at 12p13, but the origin of this material was not determined.

The add(12)(p13) abnormality is therefore an unusual cytogenetic finding whose precise biologic and pathogenic significance remains uncertain. In the context of persistent neutrophilic leukocytosis, hepatosplenomegaly, marrow granulocytic proliferation, and exclusion of secondary causes, the abnormal karyotype provides supportive evidence for an abnormal hematopoietic clone. However, the cytogenetic abnormality alone cannot establish clonality, pathogenicity, or a specific disease classification, particularly because the additional genetic material remains unidentified.

To our knowledge, an isolated ETV6-negative add(12)(p13) abnormality has not previously been reported in a CSF3R-negative neutrophilic myeloid neoplasm. This observation may represent a previously under-recognized cytogenetic abnormality; however, the novelty claim should be interpreted cautiously because the biologic significance of the abnormality remains unknown and additional cases will be required to establish whether it is recurrent or disease-associated.

The principal limitation of this case is the absence of a defining molecular abnormality and the inability to molecularly characterize the additional material at 12p13. Further molecular characterization, including techniques capable of identifying the origin of the additional chromosomal material, and long-term clinical follow-up would be valuable in determining its potential pathogenic significance.

The patient demonstrated a satisfactory hematologic response to hydroxyurea, with reduction of the leukocyte count from 68.2 × 10⁹/L to 10.12 × 10⁹/L over 90 days. This response represents a therapeutic observation and should not be interpreted as evidence supporting a particular diagnostic classification.

The clinical, morphologic, molecular, and cytogenetic findings support the presence of a BCR::ABL1-negative neutrophilic myeloid neoplasm, but the absence of a defining molecular abnormality prevents confident assignment to a specific molecularly defined entity. The case therefore illustrates the diagnostic gray zone encountered in CSF3R-negative patients with a CNL-like phenotype.

## Conclusions

We describe a rare and diagnostically challenging BCR::ABL1-negative neutrophilic myeloid neoplasm with an isolated, ETV6-negative add(12)(p13) cytogenetic abnormality in the absence of detectable canonical driver mutations. Although the patient had a clinical and morphologic phenotype suggestive of CNL, the absence of a CSF3R mutation and other canonical driver mutations precluded assignment of a definitive diagnosis of CNL. Careful consideration of aCML, CMML, MPN-U, and reactive causes of neutrophilia was therefore essential.

The isolated add(12)(p13) abnormality is an unusual cytogenetic finding that may provide supportive evidence of an abnormal hematopoietic clone in the appropriate clinicopathologic context; however, its pathogenic and disease-defining significance cannot currently be established because the additional genetic material remains unidentified and ETV6 rearrangement was not detected.

This case highlights the importance of integrating clinical findings, peripheral blood and bone marrow morphology, cytogenetics, and comprehensive molecular testing in patients with persistent neutrophilic leukocytosis. Further cases and molecular characterization of similar 12p13 abnormalities will be necessary to determine whether this finding represents a recurrent cytogenetic event or has specific biologic significance in neutrophilic myeloid neoplasms. The patient demonstrated satisfactory hematologic response to hydroxyurea over three months, with reduction of the leukocyte count from 68.2 × 10⁹/L to 10.12 × 10⁹/L. This therapeutic response is reported as a clinical outcome and does not independently establish the underlying disease classification.
